# The association between timing of initiation of antenatal care and stillbirths: a retrospective cohort study of pregnant women in Cape Town, South Africa

**DOI:** 10.1186/1471-2393-14-204

**Published:** 2014-06-13

**Authors:** Roxanne Beauclair, Greg Petro, Landon Myer

**Affiliations:** 1 The South African Department of Science and Technology/National Research Foundation (DST/NRF) Centre of Excellence in Epidemiological Modeling and Analysis (SACEMA), Stellenbosch University, c/o StIAS, Private Bag X1, Matieland, Stellenbosch 7602, South Africa; 2 Division of Epidemiology & Biostatistics, School of Public Health and Family Medicine, Faculty of Health Sciences, University of Cape Town, Cape Town, South Africa; 3 Department of Obstetrics and Gynecology, University of Cape Town and New Somerset Hospital, Cape Town, South Africa

**Keywords:** Stillbirths, Antenatal care, Gestational age, Prenatal care, South Africa

## Abstract

**Background:**

There is renewed interest in stillbirth prevention for lower-middle income countries. Early initiation of and properly timed antenatal care (ANC) is thought to reduce the risk of many adverse birth outcomes. To this end we examined if timing of the first ANC visit influences the risk of stillbirth.

**Methods:**

We conducted an analysis of a retrospective cohort of women (n = 34,671) with singleton births in a public perinatal service in Cape Town, South Africa. The main exposure was the gestational age at the first ANC visit. Bivariable analyses examining maternal characteristics by stillbirth status and gestational age at the first ANC visit, were conducted. Logistic regression, adjusting for maternal characteristics, was conducted to determine the risk of stillbirth.

**Results:**

Of the 34,671 women who initiated ANC, 27,713 women (80%) were retained until delivery. The population stillbirth rate was 4.3 per 1000 births. The adjusted models indicated there was no effect of gestational age at first ANC visit on stillbirth outcomes when analyzed as a continuous variable (aOR 1.01; 95% CI: 0.99-1.04) or in trimesters (2^nd^ Trimester aOR 0.78, 95% CI: 0.39-1.59; 3^rd^ Trimester OR 1.03, 95% CI: 0.50-2.13, both with 1^st^ Trimester as reference category). The findings were unchanged in sensitivity analyses of unobserved outcomes in non-retained women.

**Conclusion:**

The timing of a woman’s first ANC visit may not be an important determinant of stillbirths in isolation. Further research is required to examine how quality of care, incorporating established, effective biomedical interventions, influences outcomes in this setting.

## Background

In recent years the International Stillbirth Alliance has brought increased attention to stillbirths and called for renewed research on stillbirth prevention [1]. Currently stillbirths do not feature in the UN Millennium Development Goals or in the Global Burden of Disease. The lack of attention given to stillbirths in local and international arenas may be attributed to the view that stillbirths are not preventable [2]. Moreover, in many societies around the world stillbirths are often stigmatized and designated as ‘women’s rights issues’, which only serve to decrease political will for providing support and understanding to mothers and their unborn infants [2]. Worldwide there are approximately 2.65 million third-trimester stillbirths and most of the burden (98%) is in low and middle-income countries (LMICs) [3].

Antenatal conditions, such as hypertension and anemia, have been found to be strong risk factors for intrapartum complications [4]. In South Africa, from 2008–2009, 20,000 pregnancies resulted in stillbirths [5], 39% of which were intrapartum stillbirths [3]. Of mothers in South Africa who had hypertensive disease in pregnancy, 20% were associated with intrapartum stillbirths [4]. Appropriate antenatal care ANC may help to prevent some stillbirths by diagnosing and treating maternal conditions, which often endanger the fetus and mother. Moreover, early and adequate ANC has the added benefit of preparing pregnant mothers to recognize potential labor and delivery problems, as well as encouraging them to seek appropriate obstetric care during labor, which may help to prevent intrapartum stillbirths [4].

Until relatively recently the standard model of ANC in South Africa called for primigravidae women to come in for a visit a total of 12–14 times during pregnancy [6]. Recent randomized controlled trials suggest that in LMICs a schedule of ANC visits reduced to three to five visits is sufficient for mothers to have a safe delivery and give birth to healthy infants [7,8]. In one prospective, multi-site study of LMICs, mothers who did not have any ANC visits had a significant increase in risk of a stillbirth [9].

While the medical community suggests that women should present for their first ANC visit between eight and 12 weeks [10], it is not uncommon for women in Sub-Saharan Africa to begin antenatal care in the second or third trimester [11]. Studies that measure the effect of the timing of first ANC visit on birth outcomes are few and have conflicting results. In Finland, having the first ANC visit after 16 weeks has been associated with more caesarean sections, labor inductions, preterm births, as well as lower birth weights and 1-minute APGAR scores [12]. Contrary to these results, other studies that have shown that the timing of the first ANC visit has little or no effect on birth outcomes, such as birth weight [13,14].

There is reason to believe that initiating ANC early may help to prevent stillbirths in term pregnancies by preventing labor complications through early referral to skilled birth attendants, and/or by detecting and managing maternal chronic conditions (such as hypertensive disorder) and infectious diseases (such as HIV or syphilis) [15]. Given the potential importance of increasing uptake of ANC for prevention of stillbirths, the primary objective of this study was to determine if the timing of the first ANC visit influences the risk of having a stillbirth in a full-term, singleton pregnancy for a population of South African women.

## Methods

**Figure 1:**
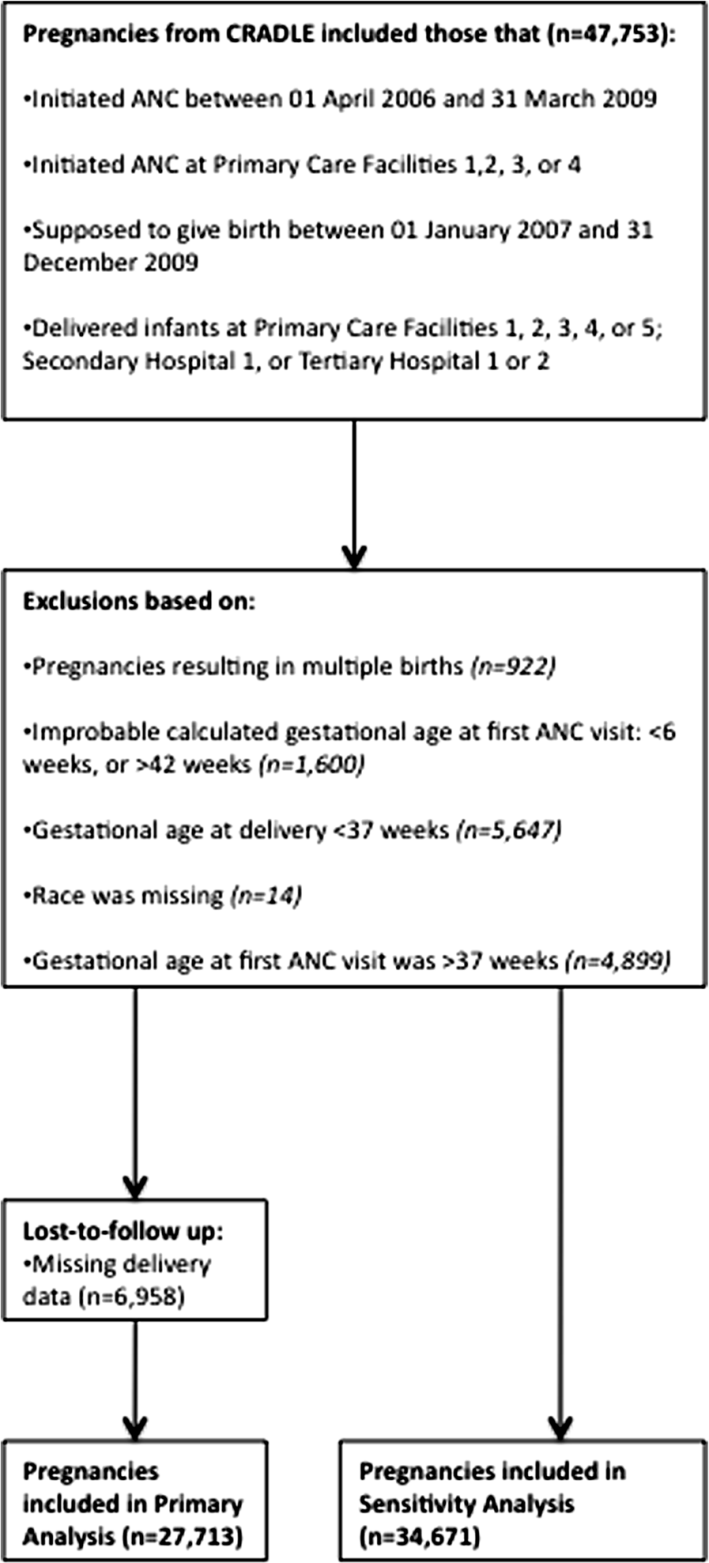
Flow chart of included and excluded women and pregnancies for the primary analysis and sensitivity analysis.

This study uses data obtained from the CRADLE database, which stores information on pregnancies for the Peninsula Maternal and Neonatal Service (PMNS). The PMNS consists of 41 different health facilities: primary level Midwife Obstetric Units, secondary and tertiary referral hospitals [16]. The PMNS is a local, public and community based perinatal service for women from predominantly poor, urban Cape Town communities. The communities are densely populated and consist of black and coloured residents who were previously disadvantaged under the old Apartheid government [17]. The Western Cape Provincial Department of Health recommends that pregnant women residing in an urban area attend a clinic before a gestational age of 20 weeks. At the first ANC visit a full initial pregnancy and general health assessment is done, including HIV testing, with appropriate counseling and referral. It is then suggested that women follow up with additional ANC visits every 6 weeks until 28 weeks, then after 34 weeks as prescribed by clinic staff. The provincial health guidelines also indicate that blood pressure and urine samples should be taken at these visits. Pregnancies regarded as high-risk due to previous labor complications, history of genetic disorders, or high blood-pressure are referred to secondary or tertiary-level hospitals for further investigations and follow-up [18].This analysis was based on a retrospective cohort of women who: 1) initiated ANC between 01 April 2006 and 31 March 2009; 2) gave birth between 01 January 2007 and 31 December 2009; and 3) initiated ANC and delivered their infants at a selected group of facilities that employed the CRADLE database. These particular pregnancies and facilities were chosen because of the high degree of completeness and presumed accuracy of CRADLE reporting at specific hospitals and time periods, but the facilities are otherwise representative of health care services in this setting. Five primary care, one secondary care, and two tertiary care facilities were chosen. Figure 1 describes the inclusion and exclusion criteria for the different analyses. Pregnancies were excluded from the analysis if they resulted in multiple births or were preterm. We focused this analysis on singleton, full-term births, as premature births and multiple pregnancies almost always result in more adverse birth outcomes. Pregnancies were excluded if the calculated gestational age at first ANC seemed highly improbable. Finally, those with missing race were excluded.

The main exposure of interest is the gestational age at first ANC visit. This variable was analyzed both as a continuous (in weeks) and categorical (in trimesters: 6–12 weeks/13-26 weeks/27-42 weeks) variable in parallel analyses. It was calculated using information in the database on the expected delivery date (EDD) at the first ANC visit. When gestation could not be calculated using an EDD based on an ultrasound, the EDD based on abdominal palpation was used. If both were missing or inaccurate, it was based on the first day of the last menstrual period (LMP). We chose to use abdominal palpation over LMP because, in this setting, LMP recall is problematic due to high prevalence of amenorrhea—from widespread use of injectable contraceptives—as well as, spotting and irregular bleeding. The only outcome variable in this study was the presence or absence of a stillbirth. We defined stillbirth as an infant that is born dead after 28 weeks of gestation [4]. However, due to our exclusion criteria we will only be reporting on stillbirths after a gestational age of 36 weeks.

Bivariable analysis was done to determine if maternal characteristics varied among those who were retained in the study and those who were lost-to-follow-up. Bivariable analysis was also conducted for maternal, first ANC visit, and delivery characteristics by gestational age at first ANC visit (trimesters) and stillbirth status. Chi-square tests of significance were used to compare frequencies in categorical variables, while the non-parametric tests, Wilcoxon rank-sum and Kruskal-Wallis rank test, were used to compare the medians of numeric variables that had two and three groups, respectively.

Two multivariable logistic regression models were fit to determine if the risk of having a stillbirth was influenced by the gestational age at first ANC visit (in continuous and trimester form). The model estimates were all adjusted for the variables: parity (nulliparous/multiparous), education level of mother (none or primary/secondary or tertiary/missing), maternal age (continuous, years), smoking status of mother (yes/no/missing), and race (other/coloured/black). These variables were chosen based upon the authors’ *a priori* causal knowledge of what was likely to influence the relationship between the gestational age at first ANC visit and the stillbirth outcome. Using causal knowledge, rather than statistical tests to construct regression models has been shown to reduce collider bias [19]. Furthermore, out of the likely candidate variables in the dataset, the variables presented here were mostly complete and presumed to be most accurate. Race was included because it is considered to be an important proxy for socio-economic status (SES) in South Africa, as it is reflects historical discrimination and continues to be predictive of the health opportunities for individuals [20]. Following on local standards, the following racial categories are used: ‘black’ African; ‘coloured’ , referring to people of mixed-race ancestry; and “Other”, referring to individuals of predominantly European Ancestry, and individuals of south Asian ancestry.

The stillbirth rate was calculated by dividing the number of stillbirths by the sum of live births and stillbirths, then multiplying the total by 1000. A sensitivity analysis was performed to see if women, who were lost-to-follow-up and met our inclusion/exclusion criteria would have affected the results if the stillbirth rate among unobserved outcomes was: a) 0 per 1,000, b) 4 per 1,000, c) 20 per 1,000 (chosen to be on par with the South African national rate [21], or d) 40 per 1,000. Stillbirths in the lost-to-follow-up population were randomly imputed. The models were constructed using multivariable logistic regression, as described above, with gestational age at first ANC analyzed continuously and in trimesters. All analyses were conducted with Stata, version 11.0 (Stata-Corp Inc., College Station, Texas, USA).

This study was approved by the institutional review board of University of Cape Town, Health Research Ethics Committee. Informed consent was not obtained per institutional review because this was a secondary analysis of routinely collected, anonymized health care data.

## Results

Overall, 27,713 women gave birth at the selected study facilities and had known details of their ANC. Another 6,958 women initiated ANC, but details of the delivery were not recorded (20.1%). Table 1 provides a detailed look at the differences in maternal and first ANC visit characteristics by lost-to-follow-up status. There were differences between the two groups for all variables except maternal age. Those that were lost-to-follow-up were more inclined to be nulliparous (46.6% vs. 43.0%), be black (38.7% vs. 33.0%), initiate ANC at Primary Care Facility 2 (50.8% vs. 24.1%), start ANC in 2006 (24.4% vs. 10.1%), and begin ANC in the first trimester (11.2% vs. 7.3).

**Table 1:** Comparison of baseline variables for those retained and those lost-to-follow-up

	**Retained**	**Lost-to-follow-up**	**p-value**
	**N (%)**	**N (%)**	
**Total**	27,713 (79.9)	6,958 (20.1)	
**Parity**			
**Primi/Multiparous**	15,795 (57.0)	3,717 (53.4)	<0.001
**Nulliparous**	11,918 (43.0)	3,241 (46.6)	
**Educational level**			
**None or primary**	1,299 (4.7)	393 (5.7)	<0.001
**Secondary or tertiary**	17,285 (62.4)	4,085 (58.7)	
**Missing**	9,129 (32.9)	2,480 (35.6)	
**Race**			
**Other**	532 (1.9)	140 (2.0)	<0.001
**Coloured**	18,046 (65.1)	4,126 (59.3)	
**Black**	9,135 (33.0)	2,692 (38.7)	
**Smoking**			
**No**	13,580 (49.0)	3,323 (47.8)	<0.001
**Yes**	6,704 (24.2)	1,441 (20.7)	
**Missing**	7,429 (26.8)	2,194 (31.5)	
**First ANC visit facility**			
**Primary care facility 1**	1,528 (5.5)	191 (2.8)	<0.001
**Primary care facility 2**	6,673 (24.1)	3,536 (50.8)	
**Primary care facility 3**	12,659 (45.7)	1,675 (24.1)	
**Primary care facility 4**	6,853 (24.7)	1,556 (22.4)	
**First ANC visit year**			
**2006**	2,788 (10.1)	1,699 (24.4)	<0.001
**2007**	10,094 (36.4)	2,663 (38.3)	
**2008**	11,946 (43.1)	1,913 (27.5)	
**2009**	2,885 (10.4)	683 (9.8)	
**Gestational age at first ANC visit (3 Categories)**			
**1**^**st **^**trimester**	2,026 (7.3)	778 (11.2)	<0.001
**2**^**nd **^**trimester**	16,641 (60.1)	4,278 (61.5)	
**3**^**rd **^**trimester**	9,046 (32.6)	1,902 (27.3)	
	**Mean (SD)**	**Mean (SD)**	
**Gestational age at first ANC visit (Continuous)**	23.0 (7.2)	21.8 (7.3)	<0.001
**Maternal age**	25.4 (6.0)	25.6 (6.2)	0.07

SD, Standard Deviation.

Only 7.3% (n = 2,026) of births had a first ANC visit take place in the first trimester—the recommended time period, while 60.1% (n = 16,641) occurred in the second trimester and 32.6% (n = 9,046) in the third trimester. Women initiating ANC in the third trimester had a tendency to be: multiparous, less educated, younger, black, and non-smoking. They also initiated ANC and delivered in Primary Care Facility 3 (Table 2). The stillbirth rate was 4.3 per 1,000 births during the study time period. In pregnancies resulting in stillbirths, the mothers were often multiparous, had no or primary education, were older, were black, were non-smokers, initiated ANC in Primary Care Facility 1 and Primary Care Facility 3, initiated ANC in 2007, and delivered in Secondary Hospital 1 and Tertiary Hospital 2 (Table 3).

**Table 2:** Maternal, first ANC visit and delivery characteristics for women who booked in the first, second and third trimesters of their pregnancy

	**Gestational age at first ANC visit**			
	**1**^**st **^**Trimester (6–12 Weeks)**	**2**^**nd **^**Trimester (13–26 Weeks)**	**3**^**rd **^**Trimester (27–42 Weeks)**	**p-value**
**Number of observations (%)**	2,026 (7.3)	16,641 (60.1)	9,046 (32.6)	
**Parity (n) (%)**				
**Nulliparous**	822 (40.6)	7,563 (45.5)	3,533 (39.1)	<0.001
**Primi/Multiparous**	1,204 (59.4)	9,078 (54.6)	5,513 (60.9)	
**Educational level (n) (%)**				
**None or primary**	99 (4.9)	706 (4.2)	494 (5.5)	<0.001
**Secondary or tertiary**	1,333 (65.8)	10,606 (63.7)	5,346 (59.1)	
**Missing**	594 (29.3)	5,329 (32.0)	3,206 (35.4)	
**Race (n) (%)**				
**Coloured**	1,641 (81.0)	11,398 (68.5)	5,007 (55.4)	<0.001
**Black**	340 (16.8)	4,943 (29.7)	3,852 (42.6)	
**Other**	45 (2.2)	300 (1.8)	187 (2.1)	
**Smoking (n) (%)**				
**Non-smoking**	968 (47.8)	8,190 (49.2)	4,422 (48.9)	<0.001
**Smokers**	591 (29.2)	4,118 (24.8)	1,995 (22.1)	
**Missing**	467 (23.1)	4,333 (26.0)	2,629 (29.1)	
**First ANC visit facility (n) (%)**				
**Primary care facility 1**	68 (3.4)	763 (4.6)	697 (7.7)	<0.001
**Primary care facility 2**	758 (37.4)	4,041 (24.3)	1,874 (20.7)	
**Primary care facility 3**	634 (31.3)	7,176 (43.1)	4,849 (53.6)	
**Primary care facility 4**	566 (27.9)	4,661 (28.0)	1,626 (18.0)	
**First ANC visit year (n) (%)**				
**2006**	390 (19.3)	1,919 (11.5)	479 (5.3)	<0.001
**2007**	654 (32.3)	5,980 (35.9)	3,460 (38.3)	
**2008**	798 (39.4)	7,031 (42.3)	4,117 (45.5)	
**2009**	184 (9.1)	1,711 (10.3)	990 (10.9)	
**Delivery facility (n) (%)**				
**Primary care facility 1**	31 (1.5)	348 (2.1)	308 (3.4)	<0.001
**Primary care facility 2**	458 (22.6)	2,779 (16.7)	1,429 (15.8)	
**Primary care facility 3**	313 (15.5)	4,105 (24.7)	3,104 (34.3)	
**Primary care facility 4**	240 (11.9)	2,509 (15.1)	949 (10.5)	
**Primary care facility 5**	2 (0.1)	24 (0.1)	12 (0.1)	
**Secondary hospital 1**	283 (14.0)	2,839 (17.1)	1,603 (17.7)	
**Tertiary hospital 1**	172 (8.5)	969 (5.82)	354 (3.9)	
**Tertiary hospital 2**	527 (26.0)	3,068 (18.4)	1,287 (14.2)	
**Maternal age**				
**Median (IQR)**	25 (22–30)	25 (21–29)	24 (21–29)	<0.001

IQR, Inter-quartile range.

**Table 3:** Maternal, first ANC visit and delivery characteristics of pregnancies that resulted in stillbirths and live births

	**Outcomes**		
	**Stillborn**	**Alive**	**p-value**
**Number of observations (%)**	119 (0.4)	27,594 (99.6)	
**Parity (n) (%)**			
**Nulliparous**	50 (42.0)	11,868 (43.0)	0.83
**Primi/Multiparous**	69 (58.0)	15,726 (57.0)	
**Educational level (n) (%)**			
**None or primary**	9 (7.6)	1,290 (4.7)	0.16
**Secondary or tertiary**	78 (65.6)	17,207 (62.4)	
**Missing**	32 (26.9)	9,097 (33.0)	
**Race (n) (%)**			
**Coloured**	56 (47.1)	17,990 (65.2)	<0.001
**Black**	59 (49.6)	9,076 (32.9)	
**Other**	4 (3.4)	528 (1.9)	
**Smoking (n) (%)**			
**Non-smoking**	71 (59.7)	13,509 (49.0)	<0.05
**Smokers**	26 (21.9)	6,678 (24.2)	
**Missing**	22 (18.5)	7,407 (26.8)	
**First ANC visit facility (n) (%)**			
**Primary care facility 1**	15 (12.6)	1,513 (5.5)	<0.01
**Primary care facility 2**	21 (17.7)	6,652 (24.1)	
**Primary care facility 3**	58 (48.7)	12,601 (45.7)	
**Primary care facility 4**	25 (21.0)	6,828 (24.7)	
**First ANC visit year (n) (%)**			
**2006**	9 (7.6)	2,779 (10.1)	0.38
**2007**	51 (42.9)	10,043 (36.4)	
**2008**	50 (42.0)	11,896 (43.1)	
**2009**	9 (7.6)	2,876 (10.4)	
**Delivery facility (n) (%)**			
**Primary care facility 1**	3 (2.5)	684 (2.5)	<0.001
**Primary care facility 2**	0 (0.0)	38 (0.1)	
**Primary care facility 3**	6 (5.0)	4,660 (16.9)	
**Primary care facility 4**	9 (7.6)	7,513 (27.2)	
**Primary care facility 5**	5 (4.2)	3,693 (13.4)	
**Secondary hospital 1**	33 (27.7)	4,692 (17.0)	
**Tertiary hospital 1**	3 (2.5)	1,492 (5.4)	
**Tertiary hospital 2**	60 (50.4)	4,822 (17.5)	
**Maternal age**			
**Median (IQR)**	26 (21–31)	25 (21–29)	0.11

IQR, Inter-quartile Range.

Table 4 demonstrates that the gestational age at first ANC visit, when analyzed continuously and in trimesters, has no significant effect on stillbirths. Although the results are not significant, each week increase of the first antenatal visit resulted in a 1% (95% CI: 0.99-1.04) increase in the odds of a stillbirth. The maternal age and race both had a significant effect on stillbirths in both analyses. Pregnancies of black women had twice the odds (aOR: 2.03; 95% CI: 1.33-2.10 and aOR: 2.01; 95% CI: 1.31-3.07, for models 1 and 2, respectively) of having a stillbirth. In both models, a one-year increase in maternal age resulted in 3% increased odds (95% CI: 1.00-1.07) of stillbirths. Finally, Table 5 contains the results of our sensitivity analysis. When the imputed stillbirth rate is 0 per 1,000, 4 per 1,000—the same as the rate in the retained population, and 20 per 1,000—the same as the South African national rate—the gestational age at first ANC visit does not increase a woman’s odds of having a stillbirth. When the stillbirth rate (40 per 1,000) is double the national rate, there is a protective effect of increased gestational age at first ANC visit (aOR 0.98; 95% CI: 0.96-0.99).

**Table 4:** Odds ratios for stillbirths and maternal characteristics using gestational age at first ANC visit in trimesters (Model 1) and as a continuous variable (Model 2)

**Variables**	**Model 1**	**Model 2**
	**aOR (95% CI)**	**aOR (95% CI)**
**Number of observations**	27,713	27,713
**Gestational age at first ANC visit (Continuous)**	--	1.01 (0.99-1.04)
**Gestational age at first ANC visit (Trimester)**		
**1**^**st **^**Trimester (ref)**	1.00	--
**2**^**nd **^**Trimester**	0.78 (0.39-1.59)	--
**3**^**rd **^**Trimester**	1.03 (0.50-2.13)	--
**Parity**		
**Nulliparous (ref)**	1.00	1.00
**Primi/Multiparous**	1.20 (0.77-1.86)	1.20 (0.77-1.86)
**Education**		
**None or primary (ref)**	1.00	1.00
**Secondary or tertiary**	0.68 (0.34-1.38)	0.68 (0.34-1.37)
**Missing**	0.77 (0.32-1.85)	0.76 (0.32-1.84)
**Race**		
**Coloured (ref)**	1.00	1.00
**Black**	2.03 (1.33-2.10)^a^	2.01 (1.31-3.07)^a^
**Other**	2.42 (0.86-6.82)	2.43 (0.86-6.83)
**Smoking**		
**Non-smoking (ref)**	1.00	1.00
**Smoking**	1.10 (0.66-1.84)	1.10 (0.66-1.84)
**Missing**	0.61 (0.30-1.25)	0.61 (0.30-1.25)
**Maternal age (continuous)**	1.03 (1.00-1.07)^a^	1.03 (1.00-1.07)^a^

All models are adjusted for parity, education, mother’s age, race, and smoking status.

aOR, Adjusted Odds Ratio.

^a^Indicates a significant aOR at 5% significance.

**Table 5:** Sensitivity analysis for stillbirths in women who were lost-to-follow-up

	**Model 1**	**Model 2**	**Model 3**	**Model 4**
	**aOR (95% CI)**	**aOR (95% CI)**	**aOR (95% CI)**	**aOR (95% CI)**
**Number of observations**	34,671	34,671	34,671	34,671
**Number of stillbirths in women lost-to-follow up**	0	28	140	279
**Total number of stillbirths**	119	147	259	398
**Gestational age at first ANC visit (Continuous)**	1.02 (0.99-1.05)	1.00 (0.98-1.03)	1.00 (0.98-1.02)	0.98 (0.96-0.99)^a^
**Gestational age at first ANC visit (Trimesters)**				
**1**^**st **^**Trimester (ref)**	1.00	1.00	1.00	1.00
**2**^**nd **^**Trimester**	0.87 (0.43-1.76)	0.81 (0.45-1.45)	0.82 (0.53-1.28)	0.60 (0.44-0.82)^a^
**3**^**rd **^**Trimester**	1.20 (0.58-2.47)	0.96 (0.52-1.78)	0.94 (0.59-1.50)	0.55 (0.39-0.78)^a^

Models 1, 2, 3, and 4 display scenarios where the assumed stillbirth rate in women who were lost-to-follow-up are: 0 per 1,000, 4 per 1,000, 20 per 1,000, and 40 per 1,000, respectively. All models are adjusted for parity, education, mother’s age, race, and smoking status.

aOR, Adjusted Odds Ratio.

^a^Indicates a significant aOR at 5% significance.

## Discussion

The results presented from this retrospective cohort of women in Cape Town, South Africa demonstrate that there appears to be no significant effect of the gestational age at first ANC visit on the odds of having a stillbirth, after adjusting for maternal characteristics. Further, the sensitivity analysis shows that even if those women who were lost-to-follow-up had stillbirths similar to the rate observed in this study or the national rate, and were included in the analysis, the finding would not change. Older mothers and women of lower SES tended to have increased odds of having a stillbirth.

Chopra et al. claim that 24% of stillbirths and neonatal deaths in South Africa could be prevented every year if families took action to prevent them by using ANC [5]. Reductions in stillbirth mortality can be achieved through ANC by increasing detection and management of hypertensive disease, fetal growth restriction and gestational diabetes as well as referring women to appropriate and skilled care for delivery when caesarean sections or inductions would be appropriate [22]. Additionally, health care providers can advise mothers on the prevention of malaria during pregnancy, prescribe folic acid supplements, test and treat syphilis [23], and encourage the use of balanced protein energy supplements [24], which are all said to improve stillbirth outcomes. Moreover, screening for congenital abnormalities as a part of ANC may help to reduce rates [24]. Authors of these studies do not always specify what ‘use of ANC’ means. The results of this study indicate that initiating ANC early, on its own, does not seem to matter so much as ensuring that some of these effective interventions take place in the antenatal period.

The stillbirth rate produced by this study, 4.3 per 1,000 births, is less than the South African national rate of 20 per 1,000 births [21]. This may be partially explained by the fact that the health care facilities in the PMNS are located in an urban area where transport to the delivery facility is more accessible and frequent, and referrals can easily be made to secondary or tertiary hospitals when complications arise [25]. Predictably, most stillbirths occurred at Secondary Hospital 1 and Tertiary Hospital 2. The lower stillbirth rate observed in Cape Town is consistent with the overall rate observed in high-income countries: less than 4 per 1,000 total births [3]. In these countries, stillbirths often result from an inability to detect and manage fetal growth restriction, maternal infections, and congenital abnormalities [3]. This suggests that the occurrence of stillbirths observed in Cape Town may be due to similar causes described in literature about high-income countries.

Alarmingly, our study also found that only 7.3% of mothers initiated ANC in the first trimester and 32.6% initiated in the third trimester. The Western Cape Provincial Department of Health advocates that women attend their first ANC session before 20 weeks to ensure that women are adequately counseled about pregnancy and screened for the possibility of having labor complications [18]. While our study does not find early initiation of ANC to be a crucial predictor of stillbirths, it may help to prevent other adverse birth outcomes [12].

Our finding that black women have increased odds of a pregnancy resulting in a stillbirth is in line with a previous finding that women from low SES households tend to have more adverse infant and child outcomes [26]. However, specific analyses and discussions about the relationship between race and stillbirths in South Africa have been deficient. Individuals of low SES in South Africa tend to reside in areas where they have decreased access to hospitals and the hospital infrastructure is inadequate and under-resourced [26]. Additionally, qualitative insights from a study done in rural South Africa found that low SES women did not perceive many hazards to pregnancy; rather the greatest perceived risks happened during labor and childbirth. One ANC visit was deemed as sufficient and was used primarily to obtain an antenatal attendance card which was required to give birth in a hospital [27]. The combination of too few ANC visits and poorer quality of infrastructure may account for the increased odds of stillbirth among women of low SES in our study, if this in turn led to decreased detection and treatment of maternal conditions that may be risk factors for stillbirths [3]. This study’s finding that increased maternal age was associated with more stillbirths corroborates results found in other countries that indicate that older mothers may be at increased risk for adverse health outcomes [28,29]. The ‘weathering hypothesis’ has been proposed to account for this. It is the idea that health starts to deteriorate in young-adulthood for women of lower SES, and the cumulative effect of ongoing disadvantages puts older mothers at higher risk [28]. Certainly, the older women of our study are from a context of high unemployment, poor access to health care, inadequate nutrition, and high levels of interpersonal and community violence, all of which would make this hypothesis plausible for this setting.

This study is the only known study to investigate how delays in first ANC visit influences the occurrence of stillbirths. Importantly, this study points to a methodological concern that arises when trying to operationalize ‘adequate’ ANC. While this study would seem to indicate that the timing of the first ANC visit does not matter for stillbirths, a more plausible scenario is that timing matters, but needs to be taken in conjunction with the number of ANC visits and content of care. A study of health care facilities in Chicago demonstrated that a majority of women utilizing ANC at these facilities had less than 80% of the recommended content during ANC. The same study also demonstrated that less adherence to recommended content was associated with more preterm births and lower birth weights [30]. Another study, conducted in Canada, indicated that health care facilities often meet recommendations for medical management of pregnancy, but neglect the advice and education component of ANC [31].

Future studies should investigate the risk of stillbirths and other birth outcomes by utilizing scoring tools that combine information on all three indicators —timing, number, and content— which are typically used independently of each other to describe ‘adequate care’. Two of the most commonly used indices to operationalize adequate care are still found to be deficient: the ‘Kessner Index’ does not look at number of ANC visits and the ‘Kotelchuck Index’ does not incorporate information on content of the visits [32]. It has been suggested that the adequacy of ANC should be operationalized with as many of the following as possible: timing of initiation of ANC, number of visits, adherence to recommended schedule, content of medical care, training of service provider, setting of care, content of educational services, and quality of the ANC system [33]. One such tool, developed in Belgium, operationalized ‘adequate’ ANC by considering whether: the first ANC visit occurred before 14 weeks; the recommended number of visits occurred at term gestation; and the appropriate number and timing of ultrasounds, blood pressure checks and blood tests were conducted [34]. A study conducted in India tested a tool like this on a population of poor and middle-income mothers and they found that a higher score resulted in more women using trained assistance at birth and safe delivery care [35].

This study has important limitations. First, the data used for the analysis was not collected and recorded by healthcare staff in a standardized way. Therefore the quality of many variables was not accurate and could not be used. Additionally, the number of total ANC visits, as well as indicators of the content of each visit (e.g. which blood tests were done), was not available, thus precluding the possibility of creating an ANC adequacy scoring tool. It is also important to note that our study only investigated stillbirths that happened after a gestational age of 36 weeks. Many of these stillbirths might have resulted from intrapartum complications that would not have been affected by the timing of ANC. Unfortunately we did not have available data on obstetric complications for the women, which has limited our understanding of the context of ANC in this study. It is, therefore, possible that early initiation of ANC in this population had an effect on antepartum stillbirths only.

Secondly, the analysis of those who were lost-to-follow-up after the first ANC visit shows that most of the maternal characteristics were differential with respect to their retention status, potentially indicating selection bias. However, our sensitivity analysis shows that even if women who were lost-to-follow-up had pregnancies resulting in stillbirths, the gestational age at first ANC visit still would not have predicted the stillbirths, unless the stillbirth rate approached double the national rate. This seems unlikely given the urban context and close proximity to good referral hospitals. Additionally, some of the largest differences occurred for the first ANC visit facility and delivery facility variables. This may be explained by the fact that some facilities were worse than others about entering delivery data into the CRADLE database, artificially making it look as though they had more lost-to-follow-up.

While our results are not generalizable to all of South Africa, they do provide insight for women with singleton, full-term births utilizing urban public hospitals in this region of South Africa. Additionally, our large sample size ensured that most of our calculations were powered enough to detect real measures of effect.

## Conclusions

The study results have implications for researchers investigating the use of ANC as a determinant of stillbirths and other birth outcomes. Future research should aim to test a combination of indicators for ‘adequate’ ANC usage. Finally, ANC messages promoted by the government and other public health professionals in South Africa should encourage clinicians to enrich their content of care and implement established effective interventions during ANC, rather than only focusing on early ANC entry.

## Competing interests

The authors declare that they have no competing interests.

## Authors’ contributions

RB, GP and LM contributed to the conception and design of the study. RB performed the statistical analysis and wrote the paper. GP and LM provided ongoing supervision and assisted with interpretation throughout the duration of the study and writing process. All three authors approved the final manuscript.

## Pre-publication history

The pre-publication history for this paper can be accessed here:

http://www.biomedcentral.com/1471-2393/14/204/prepub
